# An Overview of the Body Schema and Body Image: Theoretical Models, Methodological Settings and Pitfalls for Rehabilitation of Persons with Neurological Disorders

**DOI:** 10.3390/brainsci13101410

**Published:** 2023-10-04

**Authors:** Davide Sattin, Chiara Parma, Christian Lunetta, Aida Zulueta, Jacopo Lanzone, Luca Giani, Marta Vassallo, Mario Picozzi, Eugenio Agostino Parati

**Affiliations:** 1 Istituti Clinici Scientifici Maugeri IRCCS, Health Directorate, Via Camaldoli 64, 20138 Milan, Italy; davide.sattin@icsmaugeri.it (D.S.); mvassallo2@uninsubria.it (M.V.); 2 Istituti Clinici Scientifici Maugeri IRCCS, Neurorehabilitation Department-ALS Unit, Via Camaldoli 64, 20138 Milan, Italy; christian.lunetta@icsmaugeri.it; 3 Istituti Clinici Scientifici Maugeri IRCCS, Labion, Via Camaldoli 64, 20138 Milan, Italy; aida.zuluetamorales@icsmaugeri.it; 4 Istituti Clinici Scientifici Maugeri IRCCS, Neurorehabilitation Department, Via Camaldoli 64, 20138 Milan, Italy; jacopo.lanzone@icsmaugeri.it (J.L.); luca.giani@icsmaugeri.it (L.G.); eugenio.parati@icsmaugeri.it (E.A.P.); 5 Center for Clinical Ethics, Biotechnology and Life Sciences Department, Insubria University, 21100 Varese, Italy; mario.picozzi@uninsubria.it

**Keywords:** body schema, body image, upper limbs, action, rehabilitation

## Abstract

Given the widespread debate on the definition of the terms “Body Schema” and “Body Image”, this article presents a broad overview of the studies that have investigated the nature of these types of body representations, especially focusing on the innovative information about these two representations that could be useful for the rehabilitation of patients with different neurological disorders with motor deficits (especially those affecting the upper limbs). In particular, we analyzed (i) the different definitions and explicative models proposed, (ii) the empirical settings used to test them and (iii) the clinical and rehabilitative implications derived from the application of interventions on specific case reports. The growing number of neurological diseases with motor impairment in the general population has required the development of new rehabilitation techniques and a new phenomenological paradigm placing body schema as fundamental and intrinsic parts for action in space. In this narrative review, the focus was placed on evidence from the application of innovative rehabilitation techniques and case reports involving the upper limbs, as body parts particularly involved in finalistic voluntary actions in everyday life, discussing body representations and their functional role.

## 1. Introduction

An estimated 1 billion people live with neurological conditions, ranging from stroke and traumatic brain injury to neurodegenerative diseases [1]. After the onset of a neurological condition, patients often experience real-life challenges with movement limitations that disrupt activities of daily living and a decrease in their quality of life.

Rehabilitation protocols, defined as “a set of interventions designed to optimize functioning and reduce disability in individuals with health conditions in interaction with their environment”, are fundamental both for rehabilitating motor as well as cognitive functions and/or for decreasing the limitations due to their impairment.

In particular, considering only disability derived from upper-limb (UL) impairment, more than 75% of stroke patients remain with upper-limb impairment at the chronic stage [2,3,4], which renders the rehabilitation of the ULs after stroke a challenge. An important Cochrane review on UL rehabilitation covered 18 different types of interventions, highlighting that no high-quality evidence was available for any interventions [4]. It also showed that moderate-quality evidence indicates that constraint-induced movement therapy, mental practice, mirror therapy, interventions for sensory impairment, virtual reality and a relatively high dose of repetitive task practice could be useful, suggesting that rehabilitation protocols should probably be targeted more extensively than the classic physical approach alone.

Indeed, in recent decades, research focusing on the link between neuroscience and motor rehabilitation has developed a lot, accentuating the importance of action in constituting mental representations and vice versa. Nowadays, we know that autonomous action is characterized by different mental and motor components. Intention, sense of agency, meaning activities and muscular activities such as muscular synchronization are all parts that go beyond simple bodily movements.

Recently, new experimental methods have been developed, which allow for investigating the organization and structure of some body representations that are considered fundamental for the functional structure of movement kinematics.

In this article, we intend to use the general term “body representations” as mental representations of body parts and body-related activities. Considering that growing evidence suggests that multiple body representations exist [5], in this article, we summarize studies pointing to a dissociation between representations used for motor action and perceptual judgments and discuss how this finding can be helpful for rehabilitation.

Considering the extensive literature published after the introduction of the term “schema”, which refers to body representations from 1905 [6], and the widespread debate about the definition of the terms “body schema” and “body image”, we present an extensive overview of the published studies on these representations, analyzing (i) the different definitions and theoretical models proposed as well as (ii) the empirical settings used to test them. Finally, we discuss research perspectives derived from (iii) the critical analysis of articles describing particular misunderstood clinical case reports.

The main goal of this review is to analyze how updating the information on these two different concepts, body schema and body image, could be relevant for the rehabilitation of persons with neurological disorders (with a particular focus on those with motor impairment affecting the upper limbs). The body schema analysis can be fundamental to gain new insights for the development of innovative rehabilitation protocols.

## 2. Definitions and Nature of Body Schema and Body Image

### 2.1. Neuropsychological Taxonomies

As a framework for body schema and body image, we present the neuropsychological taxonomies, which are different models of body representation.

To distinguish between the different types of body representations, three central criteria are used [7] with different weights depending on the author:

1. Availability of consciousness (unconscious vs. conscious);
2. Functional role (action vs. perception);
3. Dynamics (short-term vs. long-term).

Cited among the best-known models are the dyadic taxonomy [8,9,10,11] and the triadic taxonomy [5,12,13]. Both describe the body schema as a sensorimotor representation of the body that is closely tied to action. The dyadic taxonomy distinguishes the body schema from the body image, with the latter conceived as a representation of the body unrelated to action, which is perceptive, conceptual or emotional in nature. Empirical support for this model can be found in the double dissociation between deafferentation (disruption of the body schema) and numbness (disruption of the body image) [10]. Triadic taxonomy, on the other hand, breaks down the body image into two different representations of the body, due to its heterogeneous and more complex nature: one of a semantic type and one of a visuospatial type. The latter, also called body structural representation (BSR) [5], corresponds to a structural description of the body and its parts, which defines the boundaries and positions of the limbs, mainly based on visual information, but also on somatic perception. The semantic representation or the body semantics (SEM) [5], on the other hand, is conceptual and linguistic in nature. In fact, it describes the categorical relationship between the parts of the body as well as their functional purpose. The dissociation between apraxia (disruption of the body schema), body-specific aphasia (disruption of body semantics) and autotopagnosia (a spatial disturbance of the body (disruption of the structural description of the body)) supports the triadic taxonomy [7].

A study by Boccia and colleagues [13] investigated the neural correlates of these three body representations in 26 patients with damage to the right hemisphere, integrating topological and hodological approaches to the analysis of the lesion deficit. The authors observed that BSR was associated with injury to the superior temporal gyrus, insula, supramarginal gyrus and temporoparietal junction, also extending to the Rolandic operculum and inferior frontal gyrus. The body schema was associated with a small cluster of voxels in the precentral and postcentral gyri, while the SEM was associated with white matter lesions at the border between the parietal and temporal lobes. These results indicate that there is a right hemisphere neuronal and connective contribution to body representation, which occurs specifically at BSR.

### 2.2. Body Schema

In recent years, various researchers have demonstrated that body representation has a multisensory nature as it is based on the integration of information from different sensory modalities (touch, proprioception, vision, vestibular signals). In the area of body representations, an important distinction between body schema and body image has historically been drawn [14], and the definition of body schema is different according to the different authors who have studied it.

The issue that seems most shared in the scientific literature is the general notion of body schema: it covers a variety of sensorimotor representations of the body that are mainly based on input information. However, de Vignemont [7] defined the body schema as a representation of posture that, based on movements or changes in position, is continuously updated, even in the absence of visual inputs, integrating information coming from peripheral receptors with that coming from muscles and joints. On the contrary, Gallagher defined body schema as a “system of sensorimotor skills that function without awareness or the need for perceptual monitoring”, contrasting it with body image described as a “system of perceptions, attitudes and beliefs related to one’s body” [9] (p. 24).

These definitions have proven to be empirically valid considering the double dissociation between patients with personal neglect and deafferented patients for example. Indeed, patients with personal neglect have problems with their perceived body image because some authors [15,16] claimed that they do not take care of the left side of their body (e.g., they do not shave or apply makeup on the left side of their face). On the other hand, deafferented patients, in the absence of tactile and proprioceptive inputs from the lower parts of the body, have a body schema damaged or replaced by a reflexive body image, since they are unable to move unless they carefully see what they are doing. By consequence, Gallagher and colleagues [9] foster a view of the body schema based on the theory of embodied cognition, such that the body schema shapes the perception that underlies cognition. The body schema, in their perspective, is an active component of body representation that integrates different positions and movements of the body in relation to the environment, thus allowing us to interact with the environment and with ourselves, and the way in which it does that structures our mind, ourselves, others and the outside world. Thus, the body schema is mainly aimed at organizing action in space and is unconscious and automatic.

However, the question of consciousness regarding body schema is still complex, controversial and far from having a clear resolution at the moment, considering that there is some experimental evidence that seems to support the idea that the body schema can be conscious in some circumstances, such as in motor imagery tasks [5], or that it could operate automatically on a subpersonal level without ever becoming conscious [9] in others (e.g., we do not need to continuously watch our limbs during movements); however, this does not mean that when we lack attention, we also lack awareness (see Sattin et al. [17], Wilterson et al. [18], Melnikoff et al. [19]).

Therefore, defining these constructs and their properties is often difficult since there are several possible ways of relating physical systems.

What seems to be accepted is that the both the body schema and the body image have a plastic and adaptable nature, as they undergo modifications over time due to the changes that our body undergoes during development. So, it seems that extra/personal “space” representations could be in continuous change as well as the “temporary” changes following multisensory stimulation (e.g., rubber hand illusion) as reported below.

### 2.3. Body Image

The notion of body image compared to that of body schema has aroused more controversy and there is no single definition to date. Body image is in fact a complex construct that includes thoughts, feelings, evaluations and behaviors related to one’s body [20]. It refers to a conscious and explicit visual representation of the way our body appears as seen from the outside in a canonical position, and it also helps us to feel the presence of a stimulus on the skin and to locate it.

According to the American Psychological Association Dictionary of Psychology, the body image is the cognitive organization of one’s appearance, including internal image, thoughts and feelings [21] that are related to body schema.

Head [22], in 1920, first defined body image as a unit of past experiences created in the cerebral sensory cortex. Like him, other authors observed a distorted or exaggerated body image from the experiment based on the body image situation of the pathological population [16]. Newell [23] saw that body image is dynamic, undergoes changes during development and also varies depending on mood or even clothing. According to Krueger, body image is the representation of identity resulting from both external and internal bodily experiences [23]. It is important to define body image because it is one of the components of personal identity.

It is the figure that one has on their own anthropometric measurements, contours and body shape, united with the feelings correlated with these factors, leading to the satisfaction or not with the body or specific parts of it [24].

As seen before, most researchers agreed to associate the body schema with the motor system, which enables action in space. In contrast, body image is identified more as representations that are not used for actions and that could be either perceptive (especially visual) or more conceptual (associated with the knowledge that one has of the different parts of the body and their usage).

In simple words, it corresponds to the way we perceive our body, similar to a 2D photograph showing a physical object and similar to how others see us from the outside. We are able to perceive our body as if it were seen not only from the outside but also from the inside from which we obtain information such as touch, proprioception and interoception. In the constitution of body image, it has been seen [25] that interoceptive processes (the sensations generated by internal organs) and interoceptive awareness may significantly contribute. Moreover, in patients with a disease, like anorexia nervosa, different manifestations of body image distortion are observable like reduced interoceptive awareness, overestimation of tactile stimuli and abnormal body scaled action.

According to some authors, body image is a multidimensional construct composed of four main components [26]:

1. Cognitive: beliefs and thoughts regarding body shape and appearance;
2. Perceptual: how we perceive the size, shape and weight of our own body and its parts;
3. Affective: feelings about the body and satisfaction or dissatisfaction;
4. Behavioral: the actions that people perform to check on, alter or cover their body, e.g., mirror checking, dieting or body avoidance (in the case of negative body image).

In this perspective, body image distortion can be considered a multidimensional symptom that comprises various elements of body image, of which the most accepted are the cognitive, the perceptive and the affective ones.

Thus, body image disturbances can be perceptual in nature (i.e., distortion) or conceptual in nature (i.e., body dissatisfaction). The first regards a failure in accurately evaluating the size of the body. Body dissatisfaction consists of negative feelings and cognitions with respect to our own body. Negative body image typically consists in a dissatisfaction of the body or its parts, which comes from a discrepancy between the perception of body image and its idealized image [24]. In a review by Kling and colleagues [27], body image is defined as a cognitive or affective evaluation of the body or appearance that a person makes, with a positive or negative valence. Even if body image studies often have a pathologizing lens, which focus on body dissatisfaction, it is nowadays increasing interesting body appreciation and positive components of body image [28].

Body image development and maintenance seem to be influenced by complex interactions between neurophysiological, sociocultural and cognitive factors [29], like gender, fashion, educational and familial influences, peer groups, evolving socialization and physical alterations [23].

Adolescence, given that it is associated with physical and social changes, is a critical period in body image development. Parents convey sociocultural and critical messages about the ideal body appearance to their sons, and the parent–adolescent relationship seems to have an important influence on the development of adolescents’ body dissatisfaction [30]. Although, in younger children, the impact of families on body image development is more significant than friends, when children become older, the role of parents decreases while the peer responses increase and become more important.

## 3. Explicative Models of Body Schema and Body Image

As seen above, body image is a complex and multi-component phenomenon; therefore, the judgments concerning it are strongly influenced by cognitive, affective, attitudinal, etc., variables and the border between body image and body schema appears fleeting sometimes.

### 3.1. Co-Construction Model

Pitron, Alsmith and de Vignemont [31] studied how body image and body schema are constructed and how they influence and reshape each other in this process of co-construction. In their model, these two bodily representations are functionally distinct and separate, but never totally independent, because their creation is partly based on their mutual interactions, hence the name of their “co-construction” model. In the case of bodily representations, action requires fine-grained spatial content in contrast to bodily experiences. With regard to body metrics, in particular, a difference in precision was observed between the two representations: the content of the body schema, since it is planned for action, is plausibly more specific and detailed unlike that of the body image, which remains more summarized. The contents of the two representations of the body often turn out to be different and in conflict, although the system remains relatively tolerant and flexible in some cases to narrow down these discrepancies (such as in Pinocchio’s illusion).

Initially, Pitron and colleagues [31] hypothesized that the process of co-construction of the two representations occurred in parallel; however, they later proposed a serial-type model, in which the representations are constructed asymmetrically. In this perspective, the body schema is the first to be constructed on the basis of multisensory signals and prior knowledge, serving as a preliminary basis for the creation of the body image. It has a more detailed spatial content that allows us to perform precise and successful movements, although it is limited because it encodes only the information about body properties needed to plan and control the action. Body image, on the other hand, integrates the information it receives from various sensory signals by giving more weight to visual inputs, and it is also influenced by social expectations about how one’s body should look or by affective factors (see above). Therefore, it is not a simple reproduction of the body schema, because, considering new inputs, it becomes more complex than the body schema, even if it loses precision and details.

In summary, the body schema prevails over the body image, but the latter has a much richer content that allows the subject to place themselves in their social world, influencing in turn the body schema in the co-construction model.

Since the two representations are distinct, the factors that contribute to their construction can determine distortions in one while keeping the other intact. Thus, although body schema influences body image, their content can become dissociated.

The authors hypothesize that there is a feedback loop between the two types of body representation; however, the influence of body image on body schema would be discontinuous and active only under certain circumstances. For example, when there is too much discrepancy between the two that persists over time, body image intervenes to recalibrate the body schema. This process could explain some data found in anorexia, in which it was seen that these patients are stuck on an “objectified body”, i.e., a body image distorted by how patients think their body is, rather than how it really is [32]. A longitudinal study [33] demonstrates how anorexia could be characterized by a body image distortion that then affects the body schema. The authors, in fact, have proposed, as the main cause of the onset and maintenance of the disorder, self-objectification, which refers to the imagination and evaluation of one’s physical appearance as if one’s body were seen as an object, i.e., as an external perspective.

The co-construction model is often able to explain both the convergence of body representations, but also their distinction in some diseases.

### 3.2. The Perception–Action Model

The Perception–Action model (dual model) highlights the functional Perception–Action distinction, explaining that the same stimulus can be processed differently depending on the task in which one is engaged. Paillard [34] applied this model to the analysis of body representations, distinguishing between “knowing where” and “knowing how to get there”. According to him, the body image is useful for making judgments about the parts of the body, through perceptual identification and recognition of the stimulus; the body schema, instead, is intended for action, and provides information on posture, limb size and strength that allow the body to move and perform actions [8,10].

In this model, the fact that two paths are identified (one dedicated to action and the other not) that operate in isolation without ever interacting has been criticized. Models such as the co-construction model affirm that it is much more likely that body schema and body image interact; in fact, in many neuropsychological disorders, there are deficits at the level of both representations, often difficult to separate and discriminate. It has been observed, for example, that ataxic patients, in some cases, can perform movements based on vision and also appear to be sensitive to some visual illusions that influence their actions [35]. It will be interesting to explore this absolute dichotomy between perception and action that the model proposes, which would elucidate, if present, the way and the level of interaction between the two types of bodily representations.

### 3.3. General Schematic Model

Slade [36,37,38] elaborated a general schematic model to summarize variables influencing body image. In his view, the body image is conceived as a free mental representation of the shape and size of the body, influenced by seven sets of factors:

- History of sensory input to body experience: throughout life, people experience varying sensory inputs about the shape, size and appearance of their bodies. This input varies over time and generates a general mental representation of the body;
- History of weight change/fluctuation;
- Cultural and social norms: some cultures encourage the goal of a thin body and this affects how we develop our attitudes about ideals of body size and shape;
- Individual attitudes to weight and shape;
- Cognitive and affective variables;
- Individual psychopathology: this clearly influences body image (e.g., anorexia and bulimia) and is certainly influenced by many of the other variables shown above (e.g., cultural and social norms, individual attitudes)
- Biological variables can influence personal body image, at least in terms of its day-to-day manifestation. One study, for example, reported a relationship between Body Integrity Identity Disorder (BIID) and the stage of the menstrual cycle [39], and a correlation between basal metabolic rate and BIID was also found [40].

## 4. Experimental Tasks: Body Schema

In the present section, we describe some tasks that are used to assess body schema in experimental settings.

*Motor imagery* (mental rotation and imagining movements): In motor imagery, one person imagines one’s body performing movements. In this sense, the imagination of a movement and the physical actions are considered fundamental parts to assess the integrity of the body schema [14,41], thus providing direct insight into action representations. So, imagery is considered to share many properties with physical movements, for example, at the kinematic level (similar physical laws) and at the neural level (shared patterns of brain activation) [42].

Settings reported in the literature using motor imagery tasks often take into consideration the variable consciousness/unconsciousness involved in motor imagery tasks considering that they can be performed consciously or unconsciously. In hand laterality judgments, for example, participants must match a visually presented hand with their own hand that they must mentally imagine and rotate [43]. This task is most often performed implicitly; however, in some cases, it may be asked to be performed explicitly without affecting performance [44]. In this last study, in particular, action representations were tested by the use of a mental rotation task involved in perceptual judgments in schizophrenic patients (which was expected to be lacking in the task both at an implicit and at an explicit level). Participants had to mentally rotate the stimulus to match the most common position, and there were three kinds of stimuli: hands, gloves and letters/numbers. For the hand stimuli, subjects had to decide whether the stimulus on the screen was a right or a left hand, by pressing keys on a computer keyboard as fast and accurately as possible. Hand condition relies only implicitly on mental imagery. For the gloves, the procedure was the same but the instruction was to explicitly imagine putting one’s own hand in the glove and to decide if it was the right or left hand. Finally, for numbers and letters, subjects were instructed to determine if the stimulus was written in a normal way or in a mirror modality. The authors described that the patients presented the same pattern of performance as control subjects but they were slower than the control group, particularly for body stimuli rather than letters, and they also showed more errors than the controls, especially for hands than letters. In the study, the authors did not find a difference in terms of reaction time or accuracy when they compared the hand condition with the gloves, both for patients and controls. For authors, this means that the hand condition depends on motor imagery itself. By contrast, a task to assess the body schema in an explicit manner could be to ask patients to imagine performing actions [5] and then mentally perform an action in a conscious way. In performing these tasks, the subjects have a conscious representation of their body while they are moving, and the body schema appears to be conscious; therefore, the availability of consciousness does not seem to be a good criterion for distinguishing body schema and body image. For the body schema in particular, it seems that the output of an action is conscious; the same cannot be said for the computations dedicated to the construction of the body schema, such as those related to multisensory integration. However, imagery and knowledge of the location of body parts in space seem to depend on information sources and, regarding this kind of argument, different theories have been proposed, distinguishing, for example, efference information that in turn predicts the location of a body part in action, the “forward model” [45,46], from the source of information regarding the body part position that is provided by feedback from sensory systems [47].

Furthermore, another interesting point is related to visual imagery tasks considering “hand” versus “hand plus other body parts” stimuli. For example, the mental rotation of hands-only, but not of hands-on-body, seems to be modulated by the stimulus view and orientation in imagery tasks. This suggests that mental rotation of hands-on-body is less dependent on biomechanical constraints and proprioceptive input and that preferential processing of visual- rather than kinesthetic-based mechanisms could be used during mental transformation of hands-on-body versus hands-only, respectively [48]. Moreover, another study on eleven stroke survivors with chronic-stage upper extremity hemiparesis in comparison to control subjects showed that area BA 6, most notably the ventral and dorsal lateral premotor cortex, was similarly activated during motor imagery and execution in both hemispheres comparing results from a task consisting of tracking a sinusoidal wave by the continuous pinching of a force transducer during both executed and imagined movements (so involving all upper-limb parties). These findings are particularly relevant because evidence in stroke patients suggests that the ipsilesional premotor cortex can be functionally reorganized to manage basic parameters of movement, a function usually assigned to M1 [49].

Another task to measure body schema is the *Pointing* to one’s body part. In order to point to body parts, we must mentally represent our body both as a target and as an effector of the action, but what is measured is not the ability to perform rapid pointing movements but only the ability to locate the body target, involving metric properties. However, without comparing them, one cannot determine whether there is a disruption in the representation of the body as a goal or as an effector. This task, therefore, is unclear and does not allow us to clearly distinguish between body schema and body image, because it may engage different types of body representations, depending on the target (e.g., one’s hand vs. a map of the hand), the type of errors measured (e.g., spatial vs. categorical) and the type of movements performed (e.g., slow gesture guided from vision or ballistic movement). As de Vignemont noted [7], pointing may involve different types of representations of the body, also considering that:

(1) To test representations of the body unrelated to actions, it is inconvenient to use an action;
(2) When performing ballistic pointing movements, it is said that the body image is not called into question. Pointing is based, in fact, on two types of bodily representation (sensorimotor and visuospatial sense, and, in some cases, also semantic).

An experimental measure should be both exclusive (specific to only one type of body representation) and exhaustive (representative of the whole-body representation and not just its parts) in order to distinguish body schema from body image. The pointing task does not have these characteristics, so it may be more appropriate, for example, to use *Reaching* and *Grasping* movements to test body schema impairments. These movements are directly related to it because we typically reach a part of the body to do something on it [7].

## 5. Experimental Tasks: Body Image

Body image research has been developed in recent years and a plethora of instruments have been designed to assess it. Kling and colleagues [27] in a systematic review tried to rigorously synthesize and evaluate body image measures to improve the cohesiveness of research in this field, in order to increase the comparability of findings and to discover the most useful and psychometrically robust instruments for research and clinical practice. They found 151 body image measures. The reason why there are many different instruments to access the body image is that it is multidimensional and the different measures allow the assessment of various components of the construct [50].

The most commonly used body image measures are those assessing a person’s evaluation of physical appearance because researchers most often have in mind a definition of body image like “how people feel about their body” [51]; in fact, body dissatisfaction measures are the most commonly used [52]. This systematic review indicates that sufficiently well-established and psychometrically robust measures exist to assess evaluative body image in various populations, like the original and revised Body Appreciation Scale (BAS and BAS-2 [53], respectively), the Body Esteem Scale for Adolescents and Adults (BESAA [54]), the Body Shape Questionnaire (BSQ [55]), the Centre for Appearance Research Valence Scale (CARVAL [56]), the Drive for Muscularity Scale (DMS [57]), the Weight and Shape Concerns (WC, SC) subscales of the EDE-Q [58], the Body Dissatisfaction subscale (BD) of the EDI-3 [59], the Appearance Evaluation subscale (AE) and Body Areas Satisfaction Scale (BASS) of the Multidimensional Body Relations Questionnaire (MBSRQ [60]). Generally, it would be appropriate to study more in-depth the psychometric properties of body image measures across genders, clinical conditions and cultural contexts, along with sexual orientations and other dimensions of identity.

However, a summary of studies included in the review highlighted that body image involves two different components [61]:

1. An attitudinal component: the feelings about own body size and shape;
2. A perceptual component: the accuracy to judge the dimensions of the body or body parts.

Evaluating the attitudinal component of body image is simpler than the perceptual component. As seen above, the most frequent measure of the attitudinal component of body image is *body dissatisfaction*, which is normally evaluated through psychometric tools. Among these tools, figure rating scales are the most commonly used. These scales contain a set of images representing bodies of different sizes ranging from underweight to overweight. Participants must choose one figure that they think represents their actual body size and another that represents how they would like to be (i.e., ideal body size). The discrepancy between the two represents dissatisfaction with the body. Eating Disorder Inventory (EDI-3) is another instrument that allows the assessment of body image. In particular, three subscales of the EDI-3 are important for measuring body image: the body dissatisfaction subscale, the drive for thinness subscale and the interoceptive awareness subscale [59].

To evaluate the perceptual component of body image, two main classes of methods have been developed:

1. *Depictive or representative methods*: these consist of comparing one’s own body to a visual or a 2D image, so the participant compares their own real body with a model image and includes tasks such as the distorting mirror [62], the distorted photograph technique [63], video distortion, template matching [61] and the silhouette [64].
2. *Metric methods*: These consist of comparing one’s own body to a physical length or a 1D standard, so the participant compares the size or shape of a body part with a non-corporeal physical standard. These methods include tasks such as the movable caliper technique [65], the image marking procedure, visual size estimation [66], the image marking procedure [67] and the adjustable light beam apparatus [61]. The image marking technique (IMT), for example, consists of an estimation and marking of various body sizes using two pens on a white sheet of paper. For each body part, the raw values (in centimeters) are calculated, which are converted into the corresponding BPI (Body Perception Index, estimated body size/real body size). The global values (that is, the total of all body parts) are then calculated for perceptual and for ideal estimations and body dissatisfaction [68].

Through these methods, commonly used in the study of eating disorders, distortions have been observed in the representation of one’s own body. In particular, with the representative methods, greater and more stable distortions [69] of the body image were observed compared to the metric ones, which suggests that the two classes of methods reflect different aspects of the body image.

Computer Generated Imagery (CGI) is a new and recent technology [29] that creates standard stimuli and personalized 3D avatars that reflect changes in body shape based on BMI, useful to test body image.

According to the triadic taxonomy, described above, body image was divided into two different components. Schwoebel and Coslett [5] designed different tasks used to test the two components. To assess the body structural representation, subjects were instructed to point to parts of their bodies that resembled pictured body parts (localization of isolated body parts), to identify the parts on a mannequin that corresponded to the part of their bodies where tactile stimuli were presented (localization of tactile input), and to identify the closest body part on the body surface to the target body part (matching body parts by localization).

Instead, body semantics was assessed through a task where subjects were asked to identify one of three pictured body parts closely related to their function (matching body parts by function) and which body part most closely corresponds to an image of clothing or a tool (matching body parts to objects and clothes).

## 6. Insights from Rehabilitation Approaches and Case Reports

**Figure 1:**
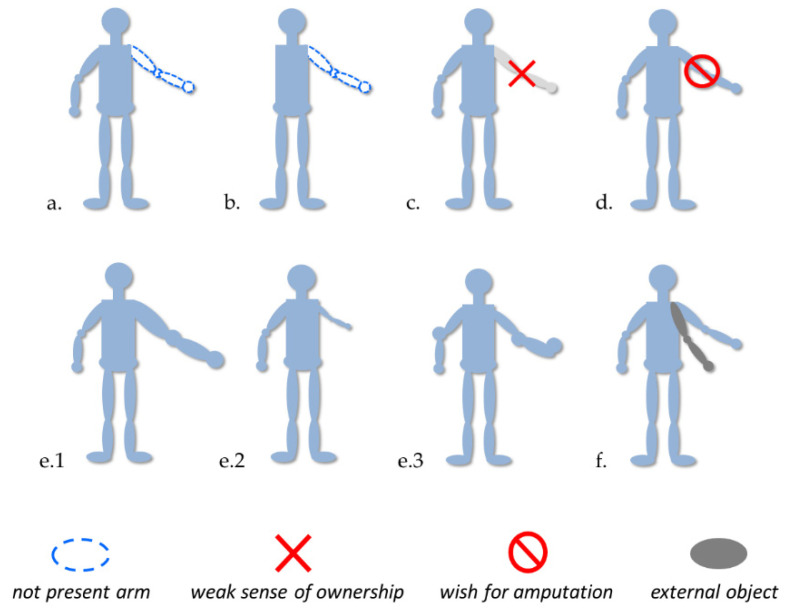
Examples of disorders probably involving body representation disfunctions. (**a**,**b**) Presence of phantom limb after amputation or aplasia; (**c**,**d**) altered sense of ownership (e.g., Body Integrity Identity Disorder, somatoparaphrenia); (**e1**,**e2**) macro/microsomatognosia, (**e3**) awareness distortion of the size, mass and shape of the body (e.g., Alice in Wonderland Syndrome); and (**f**) Supernumerary limb.

Today, we know several disorders associated with the body representation, such as phantom limbs, micro/macrosomatognosia and somatoparaphrenia (see Figure 1 for an overview). We tried to describe some particular features of these disorders and their experimental treatments useful for the generation of new insight.

### 6.1. Phantom Limb

Some patients report feeling the presence of a particular part of their body following an amputation [70] and they can sometimes also perceive pain in that part [71]. This phenomenon is called phantom limb and can affect both the upper and lower limbs. There are different types of therapeutic intervention for phantom limb pain (PLP), some of which are pharmacotherapeutic such as gabapentin, tricyclic antidepressants, morphine-based remedies, amitriptyline and ketamine [72,73] acting on different physiological systems. Others rely on non-pharmacological treatments such as transcutaneous electrical nerve stimulation (TENS), transcranial magnetic stimulation, spinal cord stimulation, use of prostheses, acupuncture and hypnosis.

*Mirror therapy* (MT) belongs to this second category and consists in mentally representing the movement, such as for motor imagery. In MT, patients are asked to use a mirror to see the reflection of the uninjured side, which results in an illusion of function in the missing area [74]. It consists of creating the visual illusion of non-painful movement in the phantom limb by reflecting the voluntary movements performed by the intact limb while looking in the mirror. The purpose of this representation of the missing limb in imaginary movement is to obtain the restoration of its projection in the corresponding cortical motor and sensory areas, thus reducing the pain related to the disruption of sensory information [75]. In fact, after an amputation, the primary somatosensory and motor cortical areas connected to the amputated limb are no longer solicited, and it has been demonstrated, using neuroimaging tools, that they are increasingly replaced by adjacent cortical areas [76]. In the medium term, MT is thought to reduce deleterious cortical reorganization [77], and the activation of (counter-lesional) mirror neurons via MT could have a pain-reducing function [75]. Indeed, MT was first proposed by Ramachandran and colleagues [78] to treat PLP, and then it was applied to other chronic pains, like neuropathic limb pain and complex regional pain syndrome.

Although its effectiveness has not yet been fully verified, MT is one of the most widely used treatments for chronic post-amputation pain. In this respect, in a review by Barbin and colleagues [79], the efficacy of MR in PLP in amputees was investigated by comparing 20 studies. They observed that, due to the considerable heterogeneity regarding MT practices and given the lack of consensus on the optimal duration of MT sessions and treatment, the efficacy of MT on PLP was not adequate enough to propose it as the first treatment [80]. It will therefore be interesting to explore new therapeutic innovations, such as somatosensory restoration through peripheral nerve stimulation, associated with the fixation of a prosthetic limb via a neural interface.

Another study of Herrador-Colmenero and colleagues [81] investigated the effectiveness of clinical interventions in treating PLP. Through virtual visual feedback therapy, it is possible to directly observe and monitor changes in the activity parameters of the injured area [82]. Using mirrors, recorded video of the intact limb, or virtual systems, the missing limb can be seen [83], and the patient is asked to synchronize their phantom limb with the observed movements [84]. This research, in particular, studied the effects of visuomotor training on motor cortex activity in patients with limb amputation. In a training program, patients learned to match voluntary “movements” of the phantom limb with prerecorded movements of a virtual hand. Before training, phantom limb movements activated the contralateral premotor cortex and, after the training, there was, in general, an increment in activity in the contralateral primary motor area. At the same time, there was a reduction in phantom pain. The authors then speculate that artificial visual feedback on the movements of the phantom limb could deceive the brain and restore the original hand and arm cortical representation.

*The mirror box technique* shows some critical issues related to the patient’s fixed position, forced to remain with the head and body turned toward the mirror [85] and concentrating on looking at the reflected image of the phantom limb, ignoring the healthy limb. The mirror box returns a symmetrical body image that is far from how our bodies appear to us. Given its statistical nature, this technique can create a temporary and restrictive illusion; however, a solution to this problem could be immersive virtual reality. When the mirror creates a symmetrical illusion, virtual reality provides us with a more appropriate and realistic image, where the asymmetrical movements of everyday life are more faithfully simulated [86].

*Virtual reality* (VR) is a technology that creates an artificial or virtual environment that simulates reality [87] and, depending on the devices used, allows different degrees of immersion and interaction with the virtual scene. VR has been used in a number of cognitive and physical rehabilitation programs. For example, VR has been shown to be an effective tool in assessing cognitive functions, such as memory and attention [88,89], and has been observed to improve physical and cognitive functions in subjects with head trauma [90]. It also produces improvements in gait and balance in individuals suffering from stroke [91], cerebral palsy [92] and spinal cord injury [93].

Immersive VR is an innovative and valuable resource that increases tolerance to pain and provides a greater ability to manage it, which derives from the sense of ownership of the avatar as one’s own body and from the perceived sense of control [94], promoting adaptive behaviors and coping strategies [95]. The embodiment effect of VR and its effectiveness in reducing pain are common to other techniques, including the mirror box technique. However, compared with the mirror box technique, VR adds a greater involvement and motivation from the user [96] and greater compliance [97].

*The motor imagery technique* is another tool used to reduce phantom limb pain, which consists in the voluntary imagination of movements in the phantom limb to compensate for the sensory deafferentation. The central nervous system activates motor actions at the level of working memory, producing a mental representation of movement in the absence of any physical body movement. The motor imagery technique compared with the mirror/virtual technique requires higher cognitive resources, and this strategy seems to be less motivating for patients, even though the brain mechanisms involved in the two techniques are similar.

Herrador-Colmeneroet al. [81], however, saw that there is still limited scientific evidence to support the effectiveness of these approaches and more robust research methods are needed. In a clinical setting, for example, it will be necessary to consider that the reduction in chronic pain depends very much on the quality of the illusion created.

Another known and consolidated therapeutic technique is *graded motor imagery (GMI)*. GMI is a variant of mirror visual feedback (MVF) that has been shown to be effective in reducing the pain and discomfort associated with movement disorders. GMI includes three phases: left/right discrimination, motor imagery exercises and mirror therapy [98,99]. Right/left discrimination is the primary goal of this treatment because it has been observed that patients with phantom limb pain, but also stroke patients with upper-limb impairment, are less accurate and slower than controls in identifying the laterality of a stimulus (e.g., left or right limb) [100,101]. This reflects the presence of a weak body schema or its alteration. Reinforcing the body schema through left/right discrimination and the explicit motor imagery technique creates a more solid basis where it is possible to intervene with the subsequent mirror therapy [102,103]. MVF and GMI, however, are based only on visual feedback or imagined actions.

Hellman and colleagues [104] hypothesized that using both GMI and artificial sensory feedback on the phantom limb, it should be possible to incorporate a neuroprosthetic or robotic system into an individual’s body schema, improving pain-related symptoms and functional performance with the prosthesis. The authors used a highly sensorized robotic artificial hand (called the “BairClaw”) to provide a combined tactile and proprioceptive feedback to BMI, producing a high-tech version of the rubber hand illusion. The Bairclaw was their testbed to incorporate a neuroprosthesis into one’s body schema and to explore the complex relationships between sensory feedback, illusion-based perceptions and body schema manipulation.

### 6.2. Aplasic Patient

It has been seen that people with a congenital absence of limbs (aplasic children) can perceive phantom limbs and are sensitive to kinematic constraints [105]. In this study, the visual experiences of two people born without arms were compared, one with and the other without phantom sensations. These participants, together with a control group, observed pictures of upper-limb movement under conditions of apparent motion. Only the aplasic individual with phantom experiences showed the same perceptual pattern as the control group; the aplasic individual without phantom sensations instead did not. This patient was born without hands and arms and so they had never executed any kind of movement with them, but they had always experienced vivid phantom limb sensations. This suggests that visual analysis of human body stimuli could have been influenced by years of experience of phantom limb movements that occurs to people with intact limbs with years of sensorimotor experience.

There are some possible explanations on how it could be possible. According to Gallagher [9], this occurs because we are all born with an innate body schema. In our point of view, however, the role of the environment in this perspective is not clear. Indeed, we all interact and see people around us who have arms, so the environment may be important in determining the existence of phantom limbs, a perspective that could be interesting to explore. Their body schema may derive from the observation of other people’s bodily movements.

However, the presence of phantom limb sensations cannot be determined only from long-term observation of other people’s limbs. If this were the case, then all sighted people with limb aplasia would describe the same sensations. Why only some people with limb aplasia experience phantom sensations remains to be elucidated.

### 6.3. Body Integrity Identity Disorder (BIID)

Body Integrity Identity Disorder is a rare disease characterized by a weak sense of ownership of one’s own body or a certain healthy limb or limbs, which causes them to be amputated to more closely represent their ideal self [106]. In BIID, there seems to be a mismatch between the body image and the body schema of certain body parts, which develops this weak sense of ownership. The only method that has been indicated to improve symptoms is surgical amputation of the alienated limbs; however, this approach has been ardently contested with arguments in favor or against it. In a study by Turbyne, de Koning, Zantvoord and Denys [106], the effects of virtual amputation in non-amputee BIID patients were investigated to determine whether or not this might affect how they experienced their BIID. Augmented reality (AR), one of the virtual technologies, was used in this study to alter the sense of body ownership in BIID patients. The participant replaces some features of the real environment with digital content that the AR shows them; however, these digital features will be experienced by them as already existing in the real world.

In their study, Turbyne and colleagues [106] observed what occurred to two patients with BIID when they were exposed to an image of their ideal self (IS) obtained via augmented reality (AR)-based stimulation that virtually amputated their alienated limbs. Upon experiencing their ideal selves, both patients reported a reduction in their complaints, suggesting that AR may have an important diagnostic and therapeutic role for BIID symptoms.

It has also been suggested that AR could be used as an adjunct to neuromodulation therapy [107] or during AR-based behavior training that aims to reintegrate the alienated limb [108]. To study the longitudinal effects of longer and repetitive exposures to the ideal self, it should also be taken into account to adapt this application for a mobile setup at home settings (e.g., portable AR glasses). In this way, it will be possible to determine the clinical utility of this method.

### 6.4. Neglect

Although rehabilitative interventions on spatial neglect and its neurophysiological origin (i.e., attentional vs. representational) is at the center of a controversial debate (see, for example, [109,110]), some case reports described in the literature offer interesting perspectives on how neglect could be considered a pervasive body representation disorder. In detail, some studies described results obtained in a patient with visual neglect that can simultaneously embody two separate, fake hands, one into the body schema and one into the body image, giving evidence in support of separable bodily representations for perception (body image) and action (body schema) that can be embodied or adapted autonomously [111,112]. In another study [113], authors found that the performance of patients with personal neglect was worse than controls in the frontal-body evocation subtest, a test where participants were asked to put tiles representing body parts on a small wooden board where only the head is depicted. On the contrary, patients’ performance was comparable in a test using an inanimate object, suggesting that performance was linked to body representations rather than the attentional system.

### 6.5. Alice in Wonderland Syndrome

The term Alice in Wonderland Syndrome (AIWS) was introduced in 1955 and indicates a group of symptoms (partially associated with migraine and epilepsy [114]) characterizing an altered body schema perception, visual, somesthetic or time illusory changes. Although microsomatognosia and macrosomatognosia (body parts are perceived to be smaller/larger than they actually are, respectively) have been described most frequently in the literature (>50% and >40% of all patients, respectively [115]), recent articles reported heterogenic symptoms. Between these, 42 were visual, 16 were somesthetic and others were non-visual symptoms [115], determining distortions of sensory perception rather than hallucinations or illusions. The association among AIWS and body schema and/or body image is still debated and, in a cognitive/motor rehabilitative perspective, there are few studies that offer data for the application of treatment for AIWS. A study using repetitive transcranial magnetic stimulation [116] in Broadman area 40, highlighted as misperception, could be determined by a synchronized activation in both auditory and visual cortices. Unfortunately, most data reported in the literature on the treatment of AIWS declare the use of a pharmacological approach (e.g., using Montelukast, Oseltamivir, Risperidone, Topiramate) often considering the presence of AIWS in relation to psychiatric disorders, especially when the duration of symptoms of AIWS tends to be long. However, symptoms’ presence tends to be short, mostly on the order of minutes to days, but they can also persist for years [117].

Therefore, AIWS presents a group of symptoms very interesting for the study of the body schema and body image, but the involvement of depersonalization, derealization, visual illusions and disorders of the perception of time (facultative symptoms of the AIWS [118]), that required an analysis of perception features and time elaboration in the brain, emphasize multidisciplinary and translational investigations to foster research knowledge and practices on AIWS.

### 6.6. Upper-Limb Rehabilitation (ULR)

Brain damage can lead to a distortion of body representations. In the literature, there are different examples of studies that tried to modulate the body distortions in patients affected by cerebral injuries through cross-modal illusions based on multisensory integration techniques, through mirror visual feedback therapy (MVFT) and the rubber hand illusion [119,120]. After stroke, negative plastic changes can occur in the brain [121]; a disuse of the upper limb after a brain lesion, for example, can lead to a reduction in the motor and sensorimotor areas of their cortical representation [122,123,124], which further compromises the limb. This phenomenon is called “learned paralysis” and can be traduced in a progressive narrowing of the representation of the affected limb in the somatosensory cortex [125]. The immobility of the limb can lead to other body distortions such as the Supernumerary phantom limb [126], typically associated with a lesion of the bilateral frontal cortex, right parietotemporal and basal ganglia [127]; anosognosia, which occurs in left brain damage [128]; and alien hand syndrome [129], which derives from damage in the motor system that causes a disturbed perception of motor actions, as well as the control of these motor actions [130].

An alteration in internal body representations, and the related distorted sense of motor awareness and motor control, is present also in patients with stroke. Such alterations in the body’s internal models could interfere with motor rehabilitation; in fact, Matamala-Gomez and colleagues [131] used a rehabilitative approach for stroke to modulate the distorted representation of the body before the conventional motor rehabilitation. For motor rehabilitation, in particular, cross-modal illusions have been used such as MVFT, which, despite evidence of effectiveness, shows some limitations in completely modifying the distorted internal representation of the paretic upper limb. Indeed, the mirror could induce an altered sense of ownership of the mirrored limb, which is seen from an inverted perspective; in fact, the limb seen in the mirror is not in line with their internal representation of the same.

Matamala-Gomez et al. [131] adopted a rehabilitation approach that employs illusions of total virtual ownership of the body, using a 360° video system with a first-person perspective, for the evaluation and modulation of the representation of the inner limb of the affected upper limb in stroke patients. The 360° system allows the complete reproduction of the distorted inner representation of the affected limb embodied in a complete virtual body before starting the rehabilitation process, which can lastly be replaced by an inner normal representation. This study offers an interesting alternative to MVFT, which is an intervention based on adequate kinesthetic and proprioceptive feedback to the upper limb before traditional motor rehabilitation.

### 6.7. Eating Disorders and Body Dysmorphic Disorder (BDD)

As explained in the previous paragraphs, the body image representation appears to be the result of a complex system of multifactorial analysis that includes active and conscious interpretation of the signals received by our sensory systems. In this case, the process of constructing this representation also seems to involve high-level processing systems with a profound involvement of cognitive processing. Thus, it is not surprising if the study of body image has evolved precisely in those sectors of medicine (i.e., neurology and psychiatry) where the processes of construction of thought have always been the subject of study.

Until recently, healthy individuals were thought to have a very accurate body image; however, recent research has shown that they have systematic distortions in body representation [132,133], which appear to be weaker forms of the distortions seen in various diseases. Disorders in which body image distortion stands out mainly include, as reported above, eating disorders [69], such as anorexia and bulimia nervosa, and Body Dysmorphic Disorder (BDD) [134]. Both disorders share the fact that those who suffer from them are dissatisfied with their body image and feel that their body, or parts of it, are unacceptable. In these cases, the common interventions and those with the greatest empirical efficacy work on the relation among thought, emotion and behavior, like Cognitive Behavioral Therapy (CBT). CBT aims to modify dysfunctional thoughts, feelings and behaviors related to body image and which contribute to the development of a negative body image [135] construction.

Self-esteem enhancing intervention is another beneficial and appropriate way to improve body image. Examples of techniques [135] are discussing alternatives to focus on appearance, and discussing individual differences and interpersonal relations.

Recently, research on body image is taking into account the neuropsychological aspects of the cognitive processing of information. Cognitive Remediation Therapy (CRT) or Cognitive Enhancement Therapy (CET) are part of a group of interventions able to improve neurocognitive abilities, like cognitive flexibility and planning, working memory, attention, set-shifting and executive functioning [136]. All these abilities are useful to facilitate rehabilitation of body image functioning in the context of medical illness in parallel to physical interventions (e.g., exercise).

Current clinical guidelines suggest, as the first-line treatment for BDD (which causes great body image distortions), CBT plus serotonin-reuptake inhibitors (SRIs). It has been seen that the SRIs help to prevent relapses. A study [137] found that 40% of a placebo group relapsed compared with only 18% of the escitalopram-continuation group, and in general, the escitalopram-continuation group made more gains. The implications of this study are that patients with BDD should remain on SRI medication for fairly long periods to avoid a relapse; in fact, available data and clinical experience show that BDD often requires SRI doses that are higher than those required to treat depression and similar to those required to treat obsessive-compulsive disorder [138]. In parallel, different medications have been suggested to treat anorexia nervosa, like selective serotonin-reuptake inhibitors, antidepressants, antipsychotics, nutritional supplementation and hormonal medications [139] with various outcomes.

Another interesting issue is that the misperception of body size and shape can be due to a bias in information processing; in fact, many cognitive biases (e.g., memory biases, attentional biases and interpretation biases) were found in patients with eating disorders. An approach recently used to assess and treat body image distortion is VR [140]. It seems to be an important alternative to motor imagery and it also allows studies that are difficult to conduct in real life. In a study [141], for example, the efficacy of a VR training program in modifying body image was investigated. Participants had to categorize a series of 3D models as thin or fat in a virtual environment. In the study, different self-report questionnaires were also administered, which tested the attitudes of the participant regarding body size/shape, weight, eating and also their tendency toward depression and self-esteem. Based on their responses, the authors found the thin/fat categorical boundary of each participant.

Participants were divided into two groups, where one was presented with the stimuli for a short period of time and the other instead had no time constraints. The authors attempted to shift the participants’ stimulus categorization toward higher BMIs by providing inflationary feedback. The study results show that VR training effectively moved the categorical boundaries of both groups of participants toward higher BMIs, unlike the control group. This effect in particular was greater for the experimental group that had no time limit in the presentation of the stimuli. Additionally, participants showed significant reductions in their concerns about body shape, weight and eating habits, which, however, were clinically significant only in the group to which the stimuli were presented for longer times. This study suggests that VR could easily be complemented by existing treatments for eating disorders.

In Table 1, we present a summary of the definitions of the two body representations, explicative models, methodologies and actual treatments used to test them.

## 7. Brain Structures and Body Representations

As easily viewed from the previous paragraphs, interest in the study of the body representation, especially for body schema, emerged from three kinds of settings in clinical rehabilitation: the first one, related to an observation of body representation distortion after a brain lesion; the second one, after a lesion/amputation of a part of the body (e.g., upper limb); and the last one, after manipulation of the environment/stimuli influencing a subject. All of these settings highlighted interesting results about the brain (and body) areas that seemed to be involved in the body representation construction, although it is difficult to think about a direct structural–functional correlation between some brain areas and the disturbance in body representation considering the multifactorial role of variables influencing this representation.

Indeed, body representations can change due to a lesion in the nervous system, as well as mental illness, with an alteration in the multisensory inner interactions of the body as a consequence [128,142]. Spatial neglect, for example, can be caused by damage in the right temporoparietal and insular areas, which disrupts spatial and bodily representations [15], creating a co-occurrence of a causal relationship. Patients with hemispatial neglect caused by right hemisphere brain damage exhibit a complex distortion of body schema (e.g., ipsilesional deviation of the median sagittal axis representation of the body or a bilateral narrowing of estimated body width [143]). In amputee or hemiparetic patients, a sensorimotor interruption following injury, arm amputation or a paresis of a body part can alter the internal representation of the body, leading to different phenomena such as phantom limb or motor anosognosia (denial of motor deficits [128]). There is also evidence that brain damage can result in distorted body representations, which can alter proprioceptive and kinesthetic signals, as well as perceptions of peripersonal space [144]. These sensory changes influence the planning, the preparation and the execution of movements, because the motor performance is continuously improved by sensorimotor circuits that constantly update internal predictions about a motor command’s outcome [46].

However, in this complex scenario, some recent studies reported interesting results about possible neural circuits involved in body schema.

During recent decades, some neuroimaging studies regarding the visual perception of the human body, as a critical component of the body schema, have identified two brain regions of the extrastriate visual cortex particularly sensitive to the perception of human bodies and body parts. These regions are the extrastriate body area (EBA), located at the posterior inferior temporal sulcus/middle temporal gyrus [145], and the fusiform body area (FBA), found ventrally in the fusiform gyrus [146,147]. Recently, it has been suggested that EBA and FBA can be functionally dissociated, with a more selective activation for local body parts in EBA relative to more holistic images of the human body in FBA [148]. It has also been seen that body schema and body structural components of body image have differential neural substrates [149], and also a neural segregation between body representations that support actions or not (non-oriented-to-action-body representation) is present [150]. The non-oriented-to-action-body representation seems to activate the somatosensory primary cortex and the supramarginal gyrus; instead, the action-oriented body representation involves the primary motor area and the right extrastriate body area.

There is substantial evidence to support that EBA and FBA are relevant to body perception; in fact, different studies show that event-related repetitive TMS applied over EBA produces a selective interruption of the perceptual tasks of the body or body parts, for example, showing difficulty in deciding whether two parts of the body are the same [151]. Besides these studies that focus on body representation in the visual processing, there is different interesting research on the role of the right posterior temporo-parietal junction (pTPJ) on embodied processing, which involves the understanding of another’s perspective, fundamental for social functioning. Experiments [152,153] with magnetoencephalography (MEG) data found that the pTPJ is a crucial hub in a wider brain network of body schema, somatosensory and motor-related areas, oscillating at the theta frequency (3–7 Hz), in the embodied perspective-taking transformations. Interfering with the right pTPJ processing using dual-pulse transcranial magnetic stimulation (dpTMS) leads to a significant reduction in embodied processing; in particular, pTPJ seems to be fundamental to transform the embodied self into another’s viewpoint, body and/or mind. Another study [154] demonstrates that right TPJ is associated with the embodied processes underpinning perspective-taking using high-definition transcranial direct current stimulation (tDCS). Through a visuospatial perspective-taking task that required understanding what another person could see or how they see it, respectively, perspective-taking (line-of-sight) was compared to perspective-taking (embodied rotation). The participant was positioned in a manner congruent or incongruent with the orientation of an avatar on a screen manipulating the embodied processing. It was observed that anodal stimulation to the right TPJ (compared to a stimulation to the dorsomedial PFC) increased the effect of the position of the body only during perspective-taking, providing evidence for a causal role for the right TPJ in the embodied component of perspective-taking.

Moreover, in a study of van Elk and colleagues [155], the activity of the right TPJ was manipulated to investigate the effects on a spatial perspective-taking task. As part of the experiment, participants were asked to mentally rotate their own bodies into the position of an avatar while undergoing either anodal, cathodal or sham transcranial direct current stimulation (tDCS) of the right TPJ. The participants were asked to judge the laterality of a stimulus feature in relation to a fixation cross on the screen as a control task. Only during anodal tDCS was a task-selective effect observed, reflected in slower reaction times following anodal rather than following cathodal and sham tDCS for the mental body transformation task, but not for the control task. This result suggests that the right TPJ plays an important role in exocentric spatial processing by impairing third-person-perspective-taking when anodal stimulation is applied.

Therefore, all these studies provide support that the right TPJ is causally involved in embodied cognitive processing relevant to social functioning.

However, the debate around the functional role of these areas is open and some authors related the activation of these neuron populations as part of a network, also involving the prefrontal areas (PFC) such as the frontopolar and dorsolateral prefrontal cortices for the generation of motor predictions, and the insular cortex (IC) that provides the convergence point for emotional and cognitive states related to the coordination between external and internal milieus, facilitating the fronto-temporal interaction in social context processing. Temporal regions (TRs) would also be integrated with feature-based information processed in frontal regions constituting fronto-insular–temporal interactions [156].

Interestingly, other data from studies that analyze visual illusions—during which people have the clear impression of seeing a second own body in the extrapersonal space, defined autoscopic phenomena and autoscopic hallucinations (see Blanke and Mohr [157] for an in-depth presentation of these phenomena)—demonstrated that such bizarre experiences result from a disturbance of multisensory integration in the right temporo-parietal cortex and in the vestibular representation in the posterior insula, though probably frontal areas and fronto-parietal connections are also involved [128].

In conclusion, the body schema seems to be at the center of neural processes that involve different brain areas, probably due to the fundamental role that it has for human movement.

## 8. Discussion

Rehabilitation protocols for persons with neurological impairment always have to do with the movements of the body in space. Whether we consider both purely physiotherapeutic protocols or protocols based more on cognitive processing, the main purposes are generally related to improving/re-enabling the execution of voluntary movements or limiting the interferences that some involuntary movements may have. This is particularly true for upper-limb rehabilitation.

In this article, we focused our attention on two body representations, like the body schema and image, as two fundamental elements that should be considered for rehabilitation protocols.

Previous results suggested that the body image and body schema are considered two representations of embodiment [16] that can work in a conscious (or unconscious) way and that seem to involve the sensorimotor information elaboration processes, the body position in space (i.e., in relation to the environment), the cognitive conceptualization of the body, and emotions and feelings toward the body (the latter especially for body image).

Considering all the components just reported, it is difficult to think about a rehabilitation protocol for movement without considering the importance of an analysis of the body representation, especially for those patients who required upper-limb rehabilitation, considering that the ULs are particularly involved in voluntary actions in everyday life.

In this discussion, we aim to analyze in-depth this latter consideration with a particular focus on rehabilitation methods derived from neuroscientific approaches, debating some clinical evidence (i.e., the effect of some rehabilitation protocols in relation to time from the acute event) and considering all the results reported in the previous paragraphs.

Firstly, we reported as some evidence that body schema could be a fundamental structure of embodiment and the idea that it could shape our perception [16].

Regarding this process of re-construction of predictions, Jeannerod [158] formulated a theory that the motor system is part of a simulation network whose function is to integrate multi-referential information, dynamically, not only to shape the motor system for preparing an action but also to provide the self with information on the feasibility and the meaning of potential actions.

Applying these considerations to evidence from clinical settings, we note that if we focus our attention on results on the functional and motor improvement of patients after stroke—for example, concentrating our analysis only on the brain, and therefore on brain plasticity after an acute event—then we can see that interventions performed early in the care process seem to favor the functional improvement by boosting the results obtained from standard rehabilitation treatments. However, using only the brain plasticity as a concern, we are unable to fully explain why other types of treatments seem instead to give greater long-term effects, such as the use of mixed protocols integrating physiotherapy with robotics (see, for example, the following meta-analysis [159]).

A possible explanation could be precisely given by the fact that the analysis of the body schema (i.e., the system that has the purpose of integrating multimodal information, including, for example, postural data) can offer a more effective simulation (and therefore optimize the results) when the patient is able to stand up, for example, or when the entire system (therefore the brain and the body) has decreased the interfering factors, the forecasting capabilities at the base of which the body schema plays a fundamental role.

As a second point, we note that in the scientific literature, different studies have demonstrated that both visual and proprioceptive feedback influences motor control, although their contributions to the online computation of body position, for example, remain unclear.

Indeed, growing evidence suggests that patients with Parkinson’s disease have faulty sensory inputs or can have physical misperceptions [160], impaired bodily sensations [161] and altered/distorted body schema [160,162,163], which also affect a variety of functions such as posture [164] with different neurophysiological hypotheses. As a practical example, studies from Dystonia reported that misperception could be determined by a time scale dysfunction. Tamura and colleagues [165] found that patients with focal hand dystonia had reduced suppression of the P27 component of the somatosensory evoked potential at the following pairs of stimuli at 5 milliseconds, but not at other short interstimulus intervals. This result was also reported by another study that found a difference between healthy controls and dystonic patients who had longer somatosensory temporal discrimination thresholds, reduced suppression of cortical and subcortical paired-pulse somatosensory evoked potentials, less spatial inhibition of simultaneous somatosensory evoked potentials and a smaller area of the early component of the high-frequency oscillations, giving particular importance to the time scale [166].

Recently, another perspective called the forward “dynamic” model has been thought to generate an estimate of the next motor state for an upcoming movement, thereby providing a dynamic representation of the current postural configuration of the body that can be utilized during movement planning and execution [167].

Parkinson and colleagues [167] found in two experiments that reaches using the right (Experiment 1) and the left (Experiment 2) arm each exhibited significantly greater BOLD responses for reaches to new target locations in an anterior region of the post-central gyrus (SPL) and in a more posterior region of the SPL, suggesting that the SPL may store a dynamically updated estimate of current limb posture, which is based upon the predicted next state estimates generated by the “forward” models used during movement planning and control. Therefore, evaluating body schema in people with PD could offer important information [168], for example, to discuss data that include asymmetries of the basal ganglia output and abnormalities in the central integration of sensory information in the analysis of causes of postural deviations that affect patients with advanced Parkinson’s disease (e.g., Pisa Syndrome), highlighting the importance of data on postural adjustments after perturbation, the loss of postural reflex and the involvement of the right posterior hypometabolism. This supports the hypothesis that PS is a result of a somatosensory perceptive deficit rather than a nigrostriatal dopaminergic imbalance [169].

Finally, there is increasing interest in how some rehabilitation techniques described above can help body schema in the integration of information after a brain lesion involving an arm. For example, the mental practice of movements, also known as motor imagery training (MIT), seems to involve the cognitive rehearsal of specific actions without overt motor output. Most stroke lesions involved subcortical regions where the lesions disrupt the anatomical connections between these areas and sensorimotor areas, and results from some studies [170] showed that the enhanced connection between ipsilesional M1 and the ipsilesional putamen might regulate voluntary motor skills and motor relearning through motor imagery training after brain damage. The improvement in upper-limb function in the MIT group is potentially related to the repair of this connection at the functional level; this suggests that the neural basis of MIT is reflected in brain activation and motor network remodeling, helping the body schema processes.

We do not know in detail how MIT or other techniques, like the MT, works in detail in the restoration of neural interactions, but the modeling processes normally used for the body schema could probably offer a perspective useful for computational analysis.

It could also be of interest to verify the difference in results obtained in patients with acquired brain lesions, like those with stroke described here or patients with disorders of consciousness, versus the results observed in patients with syndromic or genetic (like some ALS/PD/AD) diseases, in which patients somehow present and re-present expected characteristics even if not always the same, because they lie on a spectrum of disease.

In this article, we do not report operative indications for clinical rehabilitation, considering that standardized/randomized trials are few and the case reports reported results from few cases only to start a transition of these results toward the clinic setting rather than already stating some operating principles. Moreover, it is to be considered as a limitation of this article that this is not a systematic review, so the inferences allowed as well as the operative clinical indications are limited.

New perspectives for the rehabilitation and management of body representation alterations in neurological conditions can be to phenotype the set of functional representations and neuronal processes that underlie the motor act, firstly on healthy subjects and then on patients with different neurological diseases with motor impairment. In this way, it should be possible to sequence the motor act to understand in which step of the process there is an impairment, with the aim to create a treatment in conjunction or alternatively with the rehabilitation interventions already known (es MT, MIT). Moreover, it will be useful to better analyze how the processing of body–world information works in patients with sub-cortical brain lesions, giving particular attention to the analysis of configural vs. holistic modality.

In conclusion, the neuroscientific approach to rehabilitation offers new perspectives for the development of treatments for motor impairment after a neurological disease. In the present overview, we describe data on two dissociable body representations that seems to be fundamental for action and therefore for motor rehabilitation. Although we know that the framework for an explanation of complex processes involved in the management of information among neural, muscular, interoceptive, etc., systems is a challenge, the increasing number of neurological diseases with motor impairment in the general population required the development of new effective rehabilitation techniques. To achieve this aim, new phenomenological rehabilitation paradigms including body schema, especially for upper limbs that have a higher set of finalistic voluntary movements than other body parts, should be developed in the near-future.

## Figures and Tables

**Table 1:** Summary of the main characteristics and approaches to study body schema (BS) and body image (BI).

	Body Schema (BS)	Body Image (BI)
*Definitions*	Representation of posture that, based on movements or changes in position, is continuously updated, even in the absence of visual inputs, integrating information coming from peripheral receptors with that coming from muscles and joints [7]. A system of sensorimotor skills that function without awareness or the need for perceptual monitoring, contrasting it with body image described as a “system of perceptions, attitudes and beliefs related to one’s body” [8].	Cognitive organization of one’s appearance, including internal image, thoughts and feelings that are related to body schema [21]. Body image is a multidimensional construct composed of four main components [26]: Cognitive; Perceptual; Affective; Behavioral.
*Theoretical models*		General schematic model [36]
	Co-construction model [31]; The Perception–Action model for the analysis of body representations [34].	
*Experimental tasks/Assessment tools*	Motor imagery (conscious/unconscious modality); Pointing *to one’s body part;* Reaching and Grasping movements.	*Attitudinal component of BI:* Figure rating scales; Questionnaires typically about body dissatisfaction. *Perceptual component of BI:* Depictive or representative methods (e.g., distorting mirror, distorted photograph technique, video distortion, template matching); Metric methods (e.g., the movable caliper technique, visual size estimation, the image marking procedure); Computer Generated Imagery *(CGI).* *Body structural representation*: localization of isolated body parts; localization of tactile input; matching body parts by localization *Body semantics*: matching body parts by function; matching body parts to objects and clothes
*Actual non-pharmacological treatments*	Mirror therapy (MT); Virtual visual feedback therapy; Motor imagery training (MIT); Graded motor imagery (GMI); Integration of above techniques.	*For body dissatisfaction*: Cognitive Behavioral Therapy (CBT); Self-esteem-enhancing intervention; Cognitive Remediation Therapy (CRT); Cognitive Enhancement Therapy (CET).

## Data Availability

Not applicable.

## Abbreviations

List of acronyms in alphabetical order:

AIWS	Alice in Wonderland Syndrome
AR	augmented reality
BDD	Body Dysmorphic Disorder
BI	body image
BID	Body Integrity Identity Disorder
BPI	Body Perception Index
BS	body schema
BSR	body structural representation
CBT	Cognitive Behavioral Therapy
CET	Cognitive Enhancement Therapy
CGI	Computer Generated Imagery
CRT	Cognitive Remediation Therapy
EBA	extrastriate body area
FBA	fusiform body area
GMI	graded motor imagery
IC	insular cortex
IMT	image marking technique
IS	ideal self
MEG	magnetoencephalography
MIT	motor imagery training
MT	mirror therapy
MVF	mirror visual feedback
MVFT	mirror visual feedback therapy
PFC	Prefrontal Cortex
PLP	phantom limb pain
pTPJ	posterior temporo-parietal junction
SEM	body semantics
SRIs	serotonin-reuptake inhibitors
tDCS	transcranial direct current stimulation
TENS	transcutaneous electrical nerve stimulation
TMS	dual-pulse transcranial magnetic stimulation
TR	temporal region
ULR	upper-limb rehabilitation
UL	upper limb
VR	virtual reality
